# Abscess of the Liver

**Published:** 1890-12

**Authors:** Richard Douglas

**Affiliations:** Prof. of Gynecology, University of Nashville and Vanderbilt University M. B. G. S.


### Southern Medical Record.

A MONHLY JOURNAL OF MEDICINE AND SURGERY.

VOL. XX. ATLANTA, GA., DECEMBER, 1890. No. 12.

### Original Articles.

### ABSCESS OF THE LIVER.

RICHARD DOUGLAS. M. D.,

Prof. of Gynecology, University of Nashville and Vanderbilt University

M. B. G. S.

[Read at the meeting of the Tri-State Medical Association of Alabama, Georgia and Tennessee, Oct. 15, 1890, at Chattanooga Tennessee.]

Limited as we are in opportunities for study of the pathology and treatment of hepatic abscess, I feel it is presuming upon the rights of the medical men of the tropics to even discuss this subject in their absence, yet when we ascribe to their accurate observation and diagnostic acumen, our knowledge of the clinical features by which we recognize the presence of this insidious, and oftimes, most obscure condition, we will be pardoned for reviewing critically its surgical management.

"When purulent infiltration leads to liquifaction and dissolution of the tissues so that a pus-containing cavity is formed, we have what is called an abscess" (Ziegler.)

What is pus and whence it comes is ever a pertinent question in surgical pathology. It is a product generated in response to an inflammatory stimulus, which irritant, viewed from the position of modern pathology, is believed to be a bacterium acting directly, or through its agency.

Much valuable time has been expended in attempting to classify abscess of the liver, and as many varieties are given as supposed causes can be found. It would greatly simplify our labors if we would consider them all as infective, and trace, if possible, the source of sepsis. It must be admitted that we do have in the so-called tropical abscess, a suppurative process within the liver, which as yet pathologists have failed, in many cases, to associate with any lesion, or focus for infection.

Dr. Geo. Budd's view of the pathology of the so called tropical or single abscess of the liver is essentially the same as our present idea of the multiple or embolic abscess found in pyaemia (Fagge)-viz: that the abscess is the result of the absorption of some morbid product from the intestine. The strongest argument against this theory is the record of well authenticated cases, and many of them, in which death has occurred from abscess of the liver, and after the most careful search, the intestines were found to present no evidence of existing or previous inflammation.

Fagge seems to solve this problem satisfactorily in this way: "Dysentery and abscess of the liver are really common results of a like cause." It is supposed that an inflammation extends from the mucous membrane of the alimentary canal to the largest gland which opens into it," as for instance an orchitis after a urethretis.

Questionable as the pathology may be in some few cases, we, in a vast majority, can not disassociate the development of hepatic abscess with pre-existing ulceration—it is immaterial whether that be an anal fistula, sloughing pile, a dysentery, an appendicitis, or more remotely, a fractured skull. Bacteria have found a lodging, entering the blood they reach the liver, form zoogloæ, and thus is established the earliest process in the formation of an abscess. It is probable large abscesses are multiple in the beginning, and by the confluence of the small areas of suppuration, a large collection of pus formed.

The multiple abscess of pyæmia is an expression of a constitutional condition beyond the surgeon's aid. In dealing, with this subject, therefore, we will have no reference to that condition. As it is our purpose to discuss the surgical aspect of the question, we will follow up this brief pathological view with a few accepted facts for the enlightenment of those who have not paid special attention to this subject.

The general symptoms attending the formation of an abscess of the liver may be found in all the text-books, as precise and lucid as they may appear to us there, its detection and diagnosis is often very difficult, yet these difficulties may be surmounted by a "careful survey of the history, of the condition of the liver, and by exclusion of the existence of sufficient disease in other organs to account for the symptoms." Waiving then the questions of differential diagnosis by referring you to the writings of Rickman J. Godlee, F. R. C. S., Vaughn Harley, M. B., and our own Dr. Janeway, I rather direct your attention to certain circumstances and conditions attending the formation and termination of an hepatic abscess. The clinical history may present a breach which to the unwary will appear wholly incompatible with the existence of extensive suppuration. Pus formation and its attendant phenomena of chill, fever, and sweat, is an old idea so deeply ingrafted in the minds of all surgeons, we are slow to admit the existence of suppurations without such symptoms, yet it is a well known clinical fact that when an hepatic abscess is formed, and mature for opening, the temperature may be normal. "The only explanation for the absence of fever is that there is very little tension, the inflammatory material thrown out to form the abcess wall is uncommonly well defined and there is little activity around it." (Basil-Brit. Med. Jour.) To impress this important fact I will briefly report a case upon which I operated.

At the request of my friend, Dr. R. A. Hardin, I visited with him in consultation J. G. aged 22. The brief history of the case is as follows: some seven months before he passed through an attack of typhoid fever, his illness lasting about eight weeks, convalescence was slow, protracted disturbance with his bowels retarded his recovery. Some three months prior to our visit, that is about four months after the spell of typhoid fever, he had a chill followed by slight pyrexia, a trace of jaundice appearing, these symptoms passing off in a few days, his general health improved. Some two months later he complained of pain and tenderness in his right side, shortly afterwards there developed a globular swelling just beneath the margin of the ninth rib in the right hypochondriac region. The only symptoms in this case were dull heavy pain and tenderness upon pressure. There was no other general or local disturbance, absolutely no pyrexia, yet in the absence of symptoms, Dr. Hardin diagnosed abscess of the liver, which opinion I confirmed by aspiration. Having detected the existence of pus we immediately submitted the patient to an operation for its evacuation. On section the liver was found adherent to the wall of the abdomen. A free incision was made into the abscess cavity; about 8 ounces of green, viscid, inodorous pus discharged. The cavity of the abscess was irrigated with antiseptic solutions and drainage tube introduced. In four weeks all discharge had ceased, the wound closed entirely and the young man was restored to excellent general health.

We must not then, gentlemen, attach too much importance to the temperature record. To diagnose the existence of pus is the part of the medical attendant as well as the surgeon. He generally anticipates us in the care of the case and is a sharer of the responsibility of the diagnosis.

A question of much prognostic importance is the location of the abscess and the existence of adhesion. There are several methods by which the existence of adhesions may be reasonably diagnosed, such as hepatic friction audible or tactile. If on palpitation the edge of the liver remains fixed, does not move with respiration, again Wells' old idea for the detection of adhesion in ovarian tumor is practiced, the long needle introduced, the movement of the outer end will indicate whether or not the liver ascends and decends on respiration. The tension of the walls over the swelling are all signs of more or less significance. Dr. Beaver in the *Medical News*, Feb. 1890, thinks he can diagnose the existence of adhesions of the liver to parietal peritonium, "some days before classic indications of it appear." His words are: "Accompanying the first pain of peritonitis there is tenderness on pressure over the seat of pain, perhaps over an area so small that it may be covered by the tip of the finger. As the swelling of the underlying organ increases (as the abscess enlarges) the area of contact between it and the abodminal wall enlarges. The portion of peritoneum which is first inflamed becomes adherent first, and as inflammation advances the ring of adhesion extends." In expressing my admiration of the Doctor's most plausible theory, I think the opening clause of his paper—"That abscess in the liver should be evacuated as early as possible"—annuls the usefulness of his theory for the detection of adhesions. The existence or non-existence of adhesion should not influence the quick decision and conduct of the surgeon no matter how materially they affect the prognosis. It is not safe *in any case* to assume the presence of adhesions (Godlee.) Quoting again from Harley. "When an accumulation of pus has taken place in the liver it not only tends to do mischief by causing extensive disintegration of hepatic tissue, but from its becoming putrid to kill the patient by septicæmia. Therefore as a natural corollary to these facts, when pus has been detected we must evacuate it at once.

Much importance is attached by most authorities to the presence of adhesions—*of* far *greater importance* is it to the patient, that we ascertain the exact situation of the abscess. The employment of the aspirator in hepatic disorders, both as a means of diagnosis, and as a palliative measure in treatment, is no doubt practiced more frequently than one would infer from the published literature on the subject.

No authority, however, sets forth with such precision as does Dr. Harley the manner in which the instrument should be employed, the information and benefit to be gained therefrom. "A six inch long fine exploring trocar is passed up to its hilt obliquely from right to left in the liver, according to the supposed location of the abscess. The pus is then searched for by slowly and gradually withdrawing the instrument so as to allow sufficient time for a drop to appear at its orifice." If pus is found its location is noted by the depth and direction of the trocar. He further adds: "Should blood instead of pus flow, the bleeding should be encouraged. Marked benefit is likely to arise from free hepatic phlebotomy." There is much in this idea of direct bleeding by trocar from the liver substance. Dr. Harley questions if any relief can be afforded to the engorged liver by application of leeches to the abdominal wall. While his argument is founded on the strong anatomical basis of the absence of any direct vascular connection, yet in an able editorial in the "Annals of Surgery," Cathcart urges the positive clinical fact which we can all more or less confirm that marked relief of all symptoms of hepatic engorgement will follow the application of leeches to the abdomen.

This operation of freely puncturing the liver is attended by more danger than this English surgeon would lead us to think. Dr J. C. Reeves of Dayton, Ohio, diagnosed abscess of liver and in order to confirm it by aspiration, the needle was introduced into the liver. The patient immediately went into collapse, and within a minute and a half from the puncture of the needle was dead. The author thinks however that it was due to inhibition of the heart's action and not to the puncture; at all events, one must conclude that there is some risk attending hepatic puncture.

But to revert to our subject, the aspiration having proclaimed the presence of pus, what is our course? Pus here as elsewhere can only be dealt with surgically. Shall we wait for pointing? Shall we wait for adhesions? Pointing may manifest itself, so may it rupture and discharge externally, yet can we by any known means form any estimate of the tension to this abscess wall? What assurance have we that while we are waiting, Micawber-like, our abscess will not escape internally, and as for adhesions we have already discussed that point. They may never form, and if they do we can not diagnose them with certainty.

A fear of hemorrhage has led many to employ the aspirator. In such instances temporary relief alone is afforded. Pus is retained through defective drainage, consequently hectic condition and death will follow. To this fear of hemorrhage Mr. Tait himself makes confession.—"When I first attacked the liver by surgical operation I certainly was in terror of hemorrhage." A large cleft was made in the liver in freeing an adhesion to an ovarian tumor. Iron controlled the hemorrhage. In a second case he opened by an incision a large sinus, the hemorrhage was controlled by sutures, so his "respect for the liver has greatly diminished," as he quaintly puts it.

The fear of hemorrhage has led some surgeons to employ caustic or the thermocautery in the evacuation of the abscess.

These slow, painful and unscientific measures have given place to more rapid and efficient means.

The old plan of trying to cause adhesions between the liver and abdominal wall, the method of Graves,—opening by two incisions, has given place to Hepatotomy.

If an abcess is located and its evacuation determined upon, an incision should be cautiously made through the walls. We can then ascertain the condition of affairs. If the abscess is located on the surface of the liver and that viscus is adherent to the walls, the affair is quite simple, free incision, evacuation of pus, and irrigation of the cavity; provide thorough drainage and ample protective dressings and you have met all the requirements of the case. If however the abscess is in the substance of the liver and there are no adhesions, then the procedure is one of abdominal section and then fixation of the liver to the parietal wound.

To Tait belongs the chief merit of establishing the operation of hepatotomy, the entire purpose and advantages of which are to afford protection to the general peritoneal cavity from the discharging pus, and, lastly, a thorough examination of the liver can be made of second abscess before opened.

Then on carefully cutting down over the supposed seat of abscess and finding no adhesions, we should proceed as follows:

The cavity open we readily recognize the liver tissue and perhaps the abscess; soft flat sponges are packed around the field of operation inside the cavity. If necessary for diagnosis, the needle of the aspirator is thrust into the supposed site of the abscess, the pus located and with this as a guide the knife with the index finger following is introduced into the abscess cavity; holding the finger in place to prevent escape of matter each lip of the incision is seized with catch forceps, the wound is now enlarged. The first assistant raising the forceps everts the lips of the wound, the second assistant by constant pressure keeps the parietes and liver in opposition. All bleeding controlled by iron or suture as deemed best, the sac emptied of its pus and the peritoneal cavity pure and free. We are now ready to unite the liver with the parietes. This is best done by a double row of sutures, the outer row of interrupted silk sutures placed so as to interlock, thus preventing tension on the friable liver tissue in the case of much vomiting. The second row is simply the continuous whipstitch made with catgut. Rubber or glass drainage is provided and the cavity of the abscess and the dressings should be kept as nearly aseptic as possible. When an abscess is pointing upward into the pleural cavity, after resection of the rib the diaphragm is stitched to the intercostal pleura, and when that is made secure, the abscess may then be evacuated.

As illustrative of this method of primary incision I submit the following case. In June last I visited in consultation with Dr. Baskett one J. H. whole clinical history told of a long period of suffering from typhoid fever and dysentery who for the last few weeks has suffered from a large painful tender swelling in the right hypochondriac region. There was no mist of doubt surrounding this clinical history. The antecedent dysentery, the subsequent diarrhoea alternating with constipation, constant pyrexia sometimes reaching 104, occasional chill with its sweat, the great physical and mental depression were the rational symptoms, supported by a chain of physical signs making the diagnosis unquestionable, and Dr. Baskett has so proclaimed it. Not relying upon the possibility of adhesions, but preparing to do all by abdominal section and then the evacuation of the abscess, an oblique incision 4 or 5 inches length was made over point of greatest swelling. This was carried down carefully through the abdominal parietes and the liver exposed. The exploring needle was introduced and the presence of pus detected. The surface of the liver was now stitched by continuous silk suture to the parietal layer of the peritoneum, thus preventing the entrance of pus into the peritoneal cavity. Holding scalpel upon my index finger it was now thrust deep into the substance of the liver, and at least one pint of pus, blood and broken down liver tissue and detritus was evacuated. The cavity of the abscess was now thoroughouly irrigated, a large drainage tube was introduced and abundant protective dressings applied at the time of the operation, the temperature of the patient was 104, and in 12 hours was reduced to 101. With the exception of a few days when drainage was imperfect the convalescence of this case was uninterrupted. There remained for some weeks however a small inflammatory induration, which has now virtually passed away, and the recovery of the patient may be considered as complete.

